# Complete Genome Sequence of *Arthrobacter* sp. ATCC 21022, a Host for Bacteriophage Discovery

**DOI:** 10.1128/genomeA.00168-16

**Published:** 2016-03-24

**Authors:** Daniel A. Russell, Graham F. Hatfull

**Affiliations:** Department of Biological Sciences, University of Pittsburgh, Pittsburgh, Pennsylvania, USA

## Abstract

We report the complete genome sequence of *Arthrobacter* sp. ATCC 21022, a strain maintained by ATCC and a commonly used host for bacteriophage isolation and genomic analysis. The strain is prophage-free and CRISPR-free but codes for two predicted restriction-modification systems.

## GENOME ANNOUNCEMENT

*Arthrobacter* spp. are common soil bacteria classified within the family *Micrococcaceae* in the order *Actinomycetales*, and several species have potential bioremediation uses (1). Relatively few *Arthrobacter* genomes have been sequenced (12 complete, 46 whole-genome shotgun [WGS]) relative to other common soil bacterial genera, such as *Mycobacterium* (225 complete, 4,435 WGS), *Bacillus* (202 complete, 693 WGS), and *Streptomyces* (44 complete, 599 WGS), as of 2 February 2016 (2).

Here, we report the complete genome sequence of *Arthrobacter* sp. ATCC 21022, an ATCC-maintained strain. This strain is currently used for bacteriophage isolation in the Howard Hughes Medical Institute (HHMI) Science Education Alliance Phage Hunters Advancing Genomics and Evolutionary Science (SEA-PHAGES) program (3, 4), and since 2012 more than 300 individual phages have been isolated, of which more than 100 have been completely sequenced (http://phagesdb.org/hosts/strains/3).

Genomic DNA was extracted from a culture of ATCC 21022 using the Promega Wizard Genomic DNA purification kit and was sequenced by Ion Torrent on a 316 chip using 200-bp reagents. This yielded ~2.6 million reads, which were assembled into 112 contigs using Newbler version 2.6. To enhance consensus quality, an additional ~7.5 million 150-bp reads were generated using an Illumina MiSeq and aligned to the existing assembly for a total average coverage of ~350×. Where possible, contigs were joined manually using Consed version 22 (5), reducing the number of contigs to 37. These contigs were scaffolded using BLAST similarity to *Arthrobacter aurescens* TC1 (6), and contigs were then joined by performing PCR and Sanger-sequencing the products.

The genome of *Arthrobacter* sp. ATCC 21022 is a single circular molecule of 4,434,904 bp with a G+C content of 63.41%, which was annotated using the NCBI’s Prokaryotic Genome Annotation Pipeline. For bioinformatic purposes the genome was linearized with position 1 corresponding to the first base of the *dnaA* gene. The closest relative is *Arthrobacter* sp. Rue61a (7), with query coverage under standard BLASTn parameters of 76%. *Arthrobacter* sp. ATCC 21022 contains four rRNA operons and 53 tRNA genes and has 3,895 predicted protein-coding genes. We identified at least four different transposons, each of which is present in three or more locations in the genome. No CRISPR elements were identified using CRISPRFinder (8), and no intact integrated prophages were predicted using PHAST (9), although phage-related genes were identified (AUT26_17235—AUT26_17265) that are likely components of a type VI secretion system. ATCC 21022 also encodes two components of predicted restriction-modification systems (AUT26_04760 and AUT26_11520), two toxin-antitoxin systems, and a putative abortive infection protein (AUT26_01375), each of which could influence the profile of phages capable of infecting this host.

### Nucleotide sequence accession numbers.

The complete *Arthrobacter* sp. ATCC 21022 sequence and annotation are available in GenBank with the accession number CP014196. Sequencing reads have been deposited in the Sequencing Read Archive (SRA) with accession number SRP068921.
